# Study on Enhanced Oil Recovery of Nanofluid–Polymer Binary Flooding Technology in Medium-High Permeability Reservoirs

**DOI:** 10.3390/polym18020227

**Published:** 2026-01-15

**Authors:** Liqiang Yang, Xiang Peng, Qun Zhang, Liangwei Xu, Peiwen Xiao, Yuanping Lin, Yanqi Li, Chao Fang

**Affiliations:** 1 China National Petroleum Corporation, Beijing 100007, China; yangliqiang@petrochina.com.cn; 2 Key Laboratory of Nano Chemistry, State Key Laboratory of Enhanced Oil Recovery, Beijing 100083, China; 3 Oil Production Technology Research Institute of Yumen Oilfield Company, PetroChina, Jiuquan 735000, China; 4 Research Institute of Petroleum Exploration and Development, PetroChina, Beijing 100083, China; 5 Fracturing Company, Great Wall Drilling Co., Ltd., China National Petroleum Corporation (CNPC), Panjin 124010, China

**Keywords:** medium-high-permeability, nanoflooding, nanofluid–polymer binary flooding, expand sweep volume, oil recovery efficiency

## Abstract

This study investigates the application of nanofluid (iNanoW)–polymer binary flooding system to enhance oil recovery efficiency in medium-to-high permeability reservoirs. Traditional polymer flooding technologies still have the potential for further improvement in these types of reservoirs. Therefore, this study combines iNanoW with the polymer flooding system to examine its effects on the rheological properties, injectability, interfacial performance, sweep volume, and recovery factor of the polymer solution. Experimental results show that iNanoW significantly improves the injectability of the polymer solution. The introduction of iNanoW reduces the size of polymer aggregates, as demonstrated by aggregate size and rheological performance experiments. Power-law model analysis reveals that the flow behavior of the polymer solution is further improved with the introduction of iNanoW, manifested by weakened shear-thinning behavior, reduced viscosity, and optimized flowability, which in turn helps to improve oil recovery efficiency. Moreover, iNanoW interacts with polymer molecules, lowering the surface tension and enhancing wettability, thereby improving oil–water separation efficiency. Core flooding experiments show that the introduction of iNanoW significantly increases sweep volume, particularly in medium- and small-pore spaces, where oil recovery efficiencies reached 57.97% and 61.54%, respectively. These results indicate that iNanoW not only optimizes the rheological properties of the polymer solution but also improves fluid distribution during the flooding process, significantly enhancing the overall oil recovery performance. This study provides a new approach to optimizing polymer flooding technology and highlights the potential of iNanoW in improving oil recovery efficiency.

## 1. Introduction

With the increasing scarcity of global oil resources and the rising demand for energy, maximizing oil recovery has become a major challenge in the field of oil exploration and development [1,2,3,4,5,6,7]. In the later stages of development of medium-to-high permeability reservoirs, traditional waterflooding and gas flooding have limited effectiveness in improving recovery efficiency due to the gradual decline in production capacity. While these methods show some effectiveness in the early stages of development, their inefficiency gradually becomes apparent during the later stages of reservoir development. Therefore, there is an urgent need to seek new technologies to overcome the limitations of traditional methods and improve oil recovery efficiency.

Currently, Enhanced Oil Recovery (EOR) technologies have been widely applied globally, achieving certain research progress and practical success. Common chemical EOR methods include surfactant flooding, CO_2_ flooding, and polymer flooding. Surfactant flooding involves injecting surfactants into the reservoir to reduce the oil–water interfacial tension, improve oil-water separation, and enhance recovery. CO_2_ flooding involves injecting carbon dioxide gas, which dissolves in oil, reducing its viscosity and improving its mobility, thereby increasing recovery [8,9,10,11,12,13]. Polymer flooding works by increasing the viscosity of the displacement fluid, improving fluid movement and distribution within the reservoir, and expanding the sweep volume, thus improving recovery [14,15,16,17,18]. Although polymer flooding is widely used, it still faces many challenges, particularly in complex reservoir conditions or long-term development, where its effectiveness gradually diminishes [19,20,21]. Therefore, researchers have been exploring innovative technologies to improve displacement efficiency and address issues such as reservoir heterogeneity.

In recent years, with the rapid development of nanomaterials, many studies have applied them in EOR technologies to improve the performance of polymer flooding [22,23,24,25]. Nanoparticles have become a focus of attention as modifiers, improving the rheological properties of fluids and enhancing fluid permeability. Researchers’ interest in nanoparticle-polymer binary composite systems has grown, primarily exploring their potential in improving oil recovery. Existing studies have shown that nanoparticles (such as SiO_2_, Al_2_O_3_, etc.) combined with polymers can improve the injectability and displacement efficiency of polymer solutions. Salem et al. conducted a comprehensive evaluation of numerous core flooding experiments and found that the nanoparticle-polymer flooding system shows significant advantages in increasing incremental recovery, and that the properties, size, and interactions of nanoparticles with polymers significantly affect displacement performance [26].

Furthermore, numerous studies have summarized the effects of nanoparticles on polymer solution properties at both experimental and mechanistic levels. For example, one study suggested that the addition of nanoparticles can improve the rheological properties and interfacial behavior of polymer solutions, which is important for maintaining polymer performance under harsh reservoir conditions such as high salinity and high temperature [26,27,28,29,30,31]. Other works also indicate that nanoparticle-polymer fluids can promote oil–water separation by altering interfacial tension and wettability, thus improving fluid behavior during displacement [32,33,34,35].

Currently, research in this field primarily focuses on the synergistic mechanisms between different nanomaterials and polymers, system stability, and the evaluation of effectiveness under complex geological conditions. Although experimental results generally indicate that nanoparticle-polymer composite systems can significantly improve displacement performance, the size of polymer aggregates also plays a crucial role in displacement efficiency. Smaller aggregate sizes help improve the flowability and injectability of polymer solutions, thereby enhancing fluid permeability within the reservoir, expanding the sweep volume, and ultimately improving recovery [36,37,38,39]. However, research on the impact of nanoparticles on polymer aggregate sizes is still relatively limited, and the synergistic mechanism between nanoparticles and polymers requires further exploration.

In our previous research, our team discovered that iNanoW can effectively weaken the hydrogen bonding between water molecules, alter the structure of water, and reduce the size of water molecules [40,41,42,43], forming “nanowater” [6]. This enables iNanoW to penetrate small pores in low-permeability reservoirs that are not reached by conventional waterflooding, thereby increasing the sweep volume by 10–20%, which improves the recovery efficiency of low-permeability reservoirs [40,41]. This finding provides new ideas for optimizing polymer flooding. The innovation of this study lies in exploring the synergistic effect between iNanoW and polymers. Specifically, the introduction of iNanoW significantly reduces the size of polymer aggregates in the solution. Rheological analysis based on the power law model shows that the introduction of iNanoW weakens the shear thinning behavior of the polymer solution, improving its flowability and, consequently, enhancing the injectability of the solution. This increases the sweep volume of the polymer solution during displacement and improves the accessibility of fluids in the pores. Furthermore, by reducing surface tension and improving wettability, the oil–water separation efficiency is enhanced, ultimately improving oil recovery efficiency. Unlike most studies in existing literature that employ simple mixing methods, this study delves into the interaction mechanisms between polymers and nanoparticles and reveals the impact of nanoparticles on polymer aggregate sizes.

The objective of this study is to explore the application of iNanoW–polymer binary composite flooding technology in medium-to-high permeability reservoirs through experimental and theoretical research. The results show that the synergistic effect between iNanoW and polymers effectively improves the sweep volume of fluids in the pores, thereby enhancing recovery. Core flooding experiments further validate the advantages of the iNanoW–polymer binary composite flooding technology, particularly in terms of displacement efficiency in medium and small pores.

## 2. Experimental Materials and Methods

### 2.1. Materials and Equipment

The materials and instruments used in the experiments are as follows: iNanoW nanofluid (The nanoparticle concentration is 20%, silica-based modified nanomaterial, particle size: 40 ± 2 nm, viscosity: 2.0 mPa·s) and hydrolyzed polyacrylamide (HPAM) QY-7 (The polymer concentration is 34.07%, average molecular weight of 1.4 × 10^7^, emulsion polymer), purchased from Xi’an Changqing Petrochemical Corporation Co., Ltd. (Xi’an, China); The simulated oil has a viscosity of 2.0 mPa·s, consistent with the viscosity of the reservoir crude oil. It is prepared using crude oil from the Ordos Basin, China, mixed with aviation kerosene at a mass ratio of 1:5 (crude oil aviation kerosene); analytical-grade NaCl and CaCl_2_ obtained from Sinopharm Chemical Reagent Co. (Shanghai, China); artificial brine with a salinity of 4000 mg/L, containing 80 mg/L Ca^2+^, 1486 mg/L Na^+^, and 2434 mg/L Cl^−^ (The mineralization degree is derived from the mining field injection water data, viscosity: 1.0 mPa·s); high-purity deuterium water (99.9% ± 0.02%) purchased from Beijing Funuo Technology Development Co. (Beijing, China) (viscosity: 1.0 mPa·s); a 90Plus PALS dynamic light scattering analyzer (Brookhaven Instruments, Nashua, NH, USA); a HAAKE-RS600 rheometer (Thermo Fisher Scientific, Waltham, MA, USA); Attension Theta Lite multifunctional surface tensiometer (Biolin Scientific, Västra Frölunda, Sweden); and a low-field nuclear magnetic resonance (NMR) core flooding system comprising the MR-dd high-temperature/high-pressure flooding unit and the MesoMR23-060H-HTHP NMR analyzer for core characterization. The core used in this study is a medium-to-high permeability core [44,45], and the detailed core parameters are listed in Table 1.

**Figure 1:**
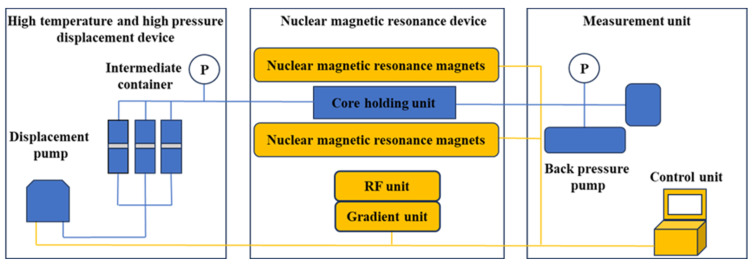
Flowchart of Low-Field Nuclear Magnetic Resonance Displacement Device.

The low-field NMR flooding system comprises two primary units: a standard flooding module and a magnetic resonance analyzer. The MR-dd high-temperature/high-pressure displacement device, manufactured by Nantong Huaxing Petroleum Instrument Co. (Nantong, China), and the MesoMR23-060H-HTHP low-field core NMR instrument, developed by Shanghai Nuomai Electronic Technology Co. (Shanghai, China), serve as the main components. A schematic diagram is shown in Figure 1. During operation, the displacing fluid and other liquids are loaded into a central reservoir, while the core sample is positioned within a specialized holder. Fluid injection is conducted through a pump system. The NMR analyzer enables real-time monitoring without halting the experiment or removing the core repeatedly.

### 2.2. Experimental Methods

#### 2.2.1. Preparation Method of iNanoW–Polymer Binary Composite Solution

The preparation of the iNanoW–polymer Binary Composite Solution is carried out as follows:

(1) An appropriate amount of iNanoW nanofluid is placed in a beaker and subjected to ultrasonic treatment for 30 min. This step ensures the proper dispersion of the nanoparticles;
(2) The total mass of the solution is set to 100 g, and the required amount of QY-7 solution is added to the beaker to achieve the desired polymer concentration;
(3) Mineral water is added to the beaker to bring the total mass of the solution to 99.5 g, and the mixture is stirred using an electric stirrer at 400 rpm for 2 h. In the preparation of the polymer solution, Brine is directly added until the total mass of the solution reaches 100 g. Once stirring is complete, the preparation is finished, and step (4) is not required;
(4) After step (1), 0.5 g of the iNanoW nanofluid is added to the beaker, and the solution is stirred at 100 rpm for 30 min with the electric stirrer, completing the preparation of the iNanoW–polymer Binary Composite Solution. Detailed quantities of each component used in the preparation of the iNanoW–polymer Binary Composite Solution can be found in Table 2.

#### 2.2.2. Evaluation of Injectability

In the MR-dd high-temperature and high-pressure flooding apparatus, the Polymer flooding/iNanoW–polymer binary composite solution was injected into the core under the conditions of a 10 MPa confining pressure and a constant pressure difference of 0.2 MPa. Fluid flow was driven by a small pressure differential, avoiding experimental errors due to compression or fluid loss. Under these conditions, the injectability of various flooding systems was evaluated. When the volume of fluid collected at the outlet reached 0.5 mL, the corresponding flow time (t) was recorded, with an experimental uncertainty of ±1 min. The flow rate (Q) was calculated using Equation (1). The flow velocity (V) of the flooding system within the core, calculated according to Equation (2), was used as a key indicator to assess injectability.

(1) $$Q=\frac{0.5\;\mathrm{mL}}{t}$$

(2) $$V=\frac{Q}{\Phi \pi R^{2}}$$

Q—flow rate, mL/min; t—flow time, min; V—flow velocity; Φ—porosity; R—core radius, cm; π—circular constant.

The injectability of the iNanoW–polymer binary composite solution, with a molecular weight of 14 million and concentrations of 500, 1000, and 1500 mg/L, is evaluated in a 150 mD core. Based on the field data of an oilfield in western China, injectability is classified as follows: (a) poor injectability is indicated by a flow velocity less than 0.014 mL/min; (b) moderate injectability corresponds to a flow velocity between 0.014 and 0.021 mL/min; (c) good injectability is characterized by a flow velocity greater than 0.021 mL/min.

#### 2.2.3. Evaluation of Polymer Aggregate Size

The 90Plus PALS dynamic light scattering particle size analyzer is used to evaluate molecular size. A solution volume of 1–2 mL in two glass cuvettes is required for each test. After placing the sample in the sample chamber, it is preheated for 5 min, with the test temperature set to 25 °C. The measurement duration is 120 s, consisting of 3 scans, with the scattering angle held at 90°. The results are statistically analyzed using the light intensity distribution method. The measurement error for particle size distribution is typically ±2–5%.

#### 2.2.4. Evaluation of Rheological Properties

The experiments are conducted using the HAAKE-RS600 rheometer, where the test temperature is maintained at 25 °C, and the PZ38 rotor model is chosen. To explore the relationship between the apparent viscosity and shear rate of the solution, the shear rate (Gp) is varied from 0.1 to 100 1/s. Based on the experimental data, the Power Law Model can be applied to describe the rheological behavior of the solution. The mathematical expression of the Power Law Model is shown in Equation (3).

(3) $$\eta =K\cdot \gamma^{n}$$

η—apparent viscosity, mPa·s; γ—shear rate, 1/s; *K*—consistency index; *n*—flow behavior index.

By performing a log-log linear fit of the experimental data, we can obtain the two key parameters of the model: the flow behavior index (*n*) and the consistency index (*K*). A value of *n* < 1 indicates shear-thinning behavior, while *n* > 1 indicates shear-thickening behavior. This analysis allows us to further explore the rheological characteristics of polymer solutions at different shear rates.

#### 2.2.5. Evaluation of Surface Tension

The surface tension of the test liquid was measured using the Attension Theta Lite multifunctional surface tensiometer, employing the drop volume method. The test solution was slowly released from a syringe, preventing the droplet from falling, and allowing a small droplet to form at the tip of the needle. The surface tension of the droplet was monitored in real-time through software, and the value before the droplet detached was recorded. The experimental error is approximately ±0.1 mN/m for surface tension measurements.

#### 2.2.6. Evaluation of Contact Angle

The contact angle was measured in this study using the Attension Theta Lite multifunctional surface tensiometer. A droplet of the test liquid was carefully placed on the surface of the rock sample, and a high-resolution camera was used to capture a side view of the droplet and the rock surface. The contact angle between the droplet and the rock surface was measured using image analysis software. The experimental error in contact angle measurement is approximately ±0.5°.

#### 2.2.7. Core Segmentation Sequence Test

**Figure 2:**
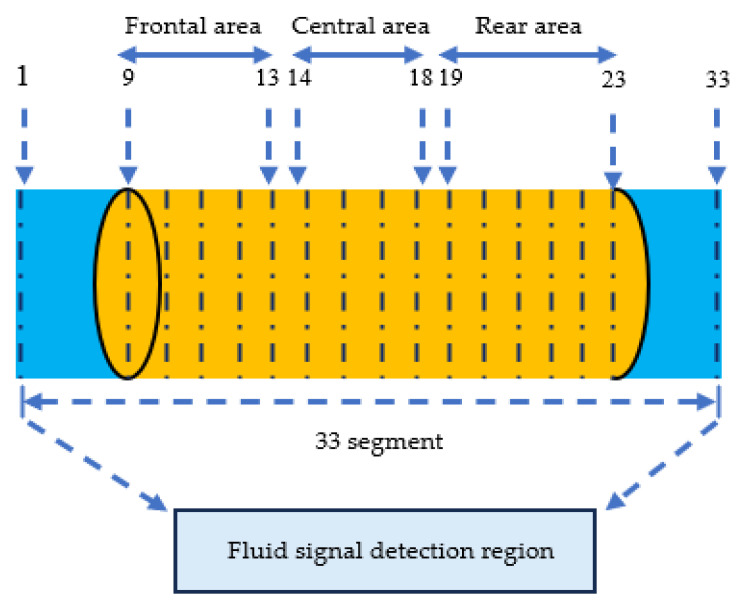
Core Segmented Sequence Test Diagram.

The MesoMR23-060H-HTHP low-field NMR analyzer was employed to conduct a segmented core scanning experiment. During this procedure, a segmented spin-echo SPI (SE-SPI) sequence was applied, with the system autonomously configuring 33 segments, The core was divided into three areas: segments 9–13 represented the frontal area, segments 14–18 the central area, and segments 19–23 the rear area. As shown in Figure 2, the apparatus partitioned the fluid signal detection region vertically into equally spaced layers, matching the number of segments in the applied sequence. NMR signal intensities corresponding to small, medium, and large pores were recorded for each segment. The principle of fluid signal acquisition remains consistent with that used in conventional *T*_2_ spectrum measurements.

#### 2.2.8. Core Flooding Experiments

**Figure 3:**
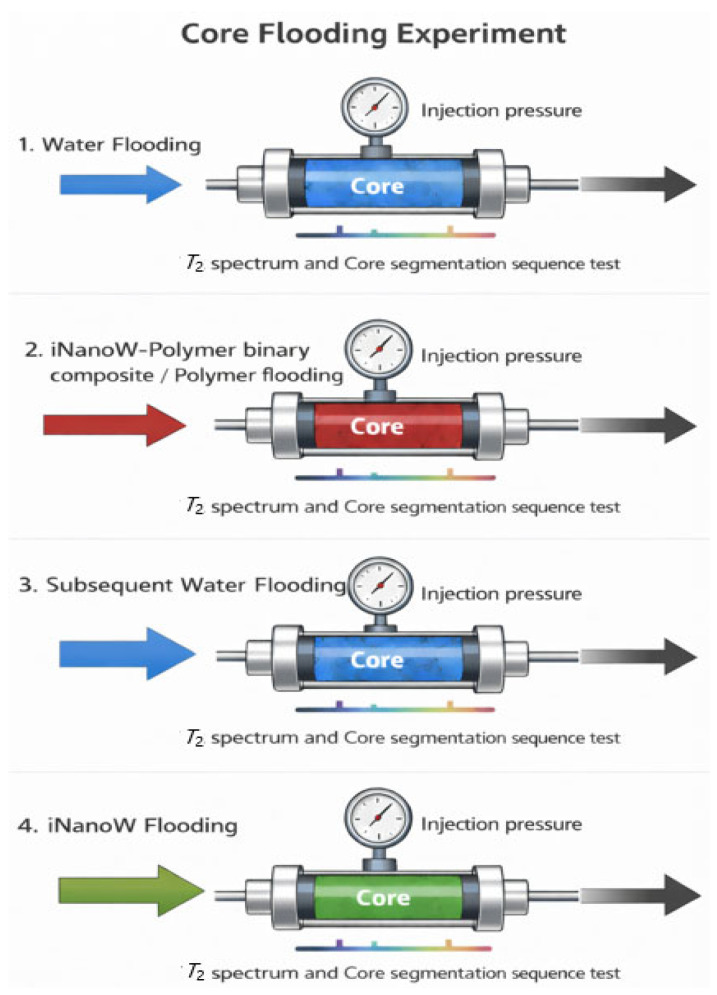
Core Flooding Experiments Flowchart.

To investigate the dynamic migration behavior of segment fluids in cores, nuclear magnetic resonance (NMR) technology was integrated with a conventional core flooding setup to perform sweep volume expansion and recovery efficiency experiments. The experimental process of core displacement is shown in Figure 3. In the sweep volume expansion experiment, segment fluids were prepared by brine (the hydrogen signal of the segment fluid can be detected by the low-field NMR equipment, and the *T*_2_ spectrum shows an increasing trend), while in the oil recovery efficiency experiment, segment fluids were prepared by brine (Deuterium water) (the hydrogen signal of the segment fluid cannot be detected by the low-field NMR equipment, and the *T*_2_ spectrum shows a decreasing trend). The methods for calculating fluid sweep volume expansion and recovery efficiency are adapted from the works of Wang et al. [42,43], and the fluid signal principles are based on the studies of Meiboom et al. [46,47,48]. The sweep volume expansion for different segment plugs was calculated using Equation (4), while oil recovery efficiency for the segment plugs was determined using Equation (5).

A polymer with a molecular weight of 14 million was chosen, and iNanoW–polymer binary composite solutions was prepared with brine, at polymer concentrations of 500, 1000, and 1500 mg/L. The iNanoW–polymer binary composite solution is based on a polymer flooding system, with iNanoW added to reach a concentration of 1000 mg/L, keeping the polymer concentration unchanged (this iNanoW concentration was derived from earlier research by the group [42]). The iNanoW concentration in the segment plugs was maintained at 1000 mg/L.

Core flooding tests were performed on cores with a permeability of 150 mD to compare and analyze the migration characteristics of various flooding solutions in cores with different permeabilities. All tests were conducted at a constant flow rate of 0.1 mL/min to maintain steady-state flooding conditions. The resistance factor (Rf) and residual resistance factor (Rff) were calculated for each stage of the injection process using Equations (6) and (7).

(4) $$S=\frac{S_{p}-S_{w}}{S_{w}-S_{c}}\times 100\%$$

(5) $$R=\frac{S_{w}-S_{p}}{S_{o}-S_{c}}\times 100\%$$

S—Improvement of sweep efficiency; R—Enhanced oil recovery; S_w_—Stable *T*_2_ spectrum peak area of water flooding; S_p_—Stable *T*_2_ spectrum peak area of iNanoW flooding, iNanoW–polymer binary flooding or subsequent water flooding; S_c_—*T*_2_ spectrum peak area of core saturated deuterium water; S_o_—*T*_2_ spectrum peak area of core saturated oil.

(6) $$\mathrm{Rf}=\frac{P_{P}}{P_{w}}$$

(7) $$\mathrm{Rff}=\frac{P_{s}}{P_{w}}$$

Rf—resistance factor; Rff—residual resistance factor; P_w_—water flooding segment plugs stable pressure; P_p_—Polymer flooding/iNanoW–polymer binary flooding segment plugs stable pressure; P_s_—subsequent water flooding segment plugs stable pressure.

## 3. Results and Discussion

### 3.1. Effect of iNanoW on Polymer Flooding Injectability

The study indicates that in unconventional medium-to high-permeability reservoirs [44,45], the injectability of a polymer solution is strongly dependent on both polymer concentration and molecular structure. As polymer concentration increases, the number of entangled polymer chains and the size of aggregates increase, leading to higher solution viscosity and greater flow resistance. This phenomenon is reflected in the measured data, where higher polymer concentrations correspond to lower flow velocities and seepage rates.

The flow velocity of the iNanoW–polymer binary composite solution with polymer concentrations of 500, 1000, and 1500 mg/L (with iNanoW concentration of 1000 mg/L) in a 150 mD core was measured. The results, shown in Table 3, indicate that the flow velocity of the polymer solution in the core is significantly influenced by its concentration. Under the same polymer concentration, the flow velocity of the iNanoW–polymer binary composite solution is higher than that of the pure polymer flooding, indicating better injectability. Therefore, the iNanoW–polymer binary composite solution with varying injectability was selected as the focus of this study.

The addition of iNanoW mitigates this effect by reducing polymer aggregation. Smaller, more uniformly distributed polymer molecules exhibit lower hydrodynamic resistance, allowing the solution to flow more easily through the porous medium. As a result, even at higher polymer concentrations, the iNanoW–polymer composite system maintains relatively higher flow velocities compared with pure polymer flooding. This enhanced injectability ensures that the polymer solution can penetrate the core more effectively, reducing the risk of injection blockage and early breakthrough, which is particularly important for high-concentration polymer flooding where viscosity-induced flow hindrance is pronounced.

Moreover, the experimental results suggest a concentration-dependent improvement in injectability (shown in Table 3): at low polymer concentrations (500 mg/L), both systems show good injectability, while at medium (1000 mg/L) and high concentrations (1500 mg/L), the flow velocity decreases, yet the iNanoW–polymer system consistently outperforms the pure polymer system. This demonstrates that iNanoW not only improves flow performance under challenging high-viscosity conditions but also broadens the operational window for polymer flooding, allowing higher polymer concentrations to be used without severely compromising injectability.

In summary, the inclusion of iNanoW enhances the injectability of polymer solutions by reducing aggregate size and viscosity-related flow resistance, ensuring more uniform and efficient displacement in the porous medium. These properties make the iNanoW–polymer binary system particularly advantageous for core flooding experiments and potential field-scale applications.

### 3.2. Effect of iNanoW on Polymer Aggregate Size

The observation of bimodal peaks in the dynamic light scattering (DLS) data typically indicates the presence of two distinct populations of aggregates in the solution. This phenomenon suggests a polydispersity in the size distribution of the polymer aggregates, which may not follow a uniform distribution. The dual peaks likely result from the intermolecular interactions and entanglement between polymer chains, which form aggregates of varying sizes. Furthermore, this bimodal distribution can be interpreted in terms of aggregation dynamics, where one peak corresponds to smaller aggregates (possibly formed by lower molecular weight chains or those with fewer entanglements), and the other represents larger, more entangled polymer aggregates. This dual-peak structure accounts for the broader size distribution observed in the DLS data.

**Figure 4:**
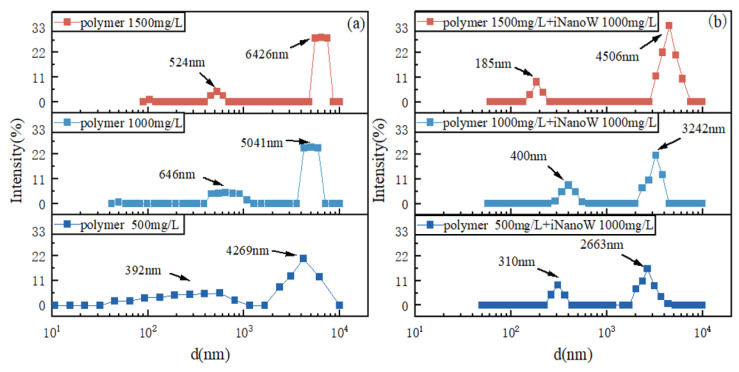
(**a**) Aggregate Molecular Size in Polymer Solution; (**b**) Aggregate Molecular Size in iNanoW–polymer binary composite Solution.

As the polymer concentration increases (from 1000 mg/L to 2000 mg/L), intermolecular interactions, particularly hydrogen bonding between polymer chains, become stronger, leading to a significant increase in the size of the polymer aggregates. This is clearly reflected in the DLS spectra [49,50,51], where the peaks shift toward larger sizes, and the width of the peaks increases (as shown in Figure 4a). Higher polymer concentrations enhance the entanglement of polymer chains, promoting the formation of larger aggregates. This effect is most pronounced in the 2000 mg/L polymer solution, which exhibits significantly larger aggregate sizes compared to the 1000 mg/L and 1500 mg/L concentrations.

The addition of iNanoW to the polymer solution (as shown in Figure 4b) leads to a marked reduction in the size distribution of the aggregates. This suggests that iNanoW effectively disrupts the hydrogen bonding between polymer chains, causing the polymer chains to “unwind” or become less entangled, which results in a reduction in the size of the aggregates. Specifically, at the same polymer concentration, the presence of iNanoW reduces the size of the aggregates by approximately 29% to 37%. This observation further supports the notion that iNanoW interferes with the intermolecular hydrogen bonding, weakening the interactions between polymer chains and thereby reducing the size of the polymer aggregates, which results in a more uniform distribution of aggregate sizes in the solution.

Rodriguez-Loya et al. [52], proposed that the incorporation of nanoparticles can substantially influence the aggregation dynamics of polymer and colloidal particles. Dynamic light scattering (DLS) is not only employed to assess particle size distribution, but also to monitor and regulate the aggregation behavior in systems that include both nanoparticles and polymers. This suggests that the presence of additives can modify intermolecular interactions, ultimately leading to a reduction in the size distribution of the aggregates.

Furthermore, Sun et al. [32], suggested that the introduction of nanoparticles into polymer matrices may disrupt the interactions and mobility of polymer chains, consequently influencing the formation of aggregates. Specifically, when nanoparticles are positioned between polymer chains, they can alter the internal friction and overall dynamic behavior of the chains. This phenomenon is consistent with the observed reduction in aggregate size in our system upon the incorporation of iNanoW.

### 3.3. Effect of iNanoW on Polymer Solution Viscosity

**Figure 5:**
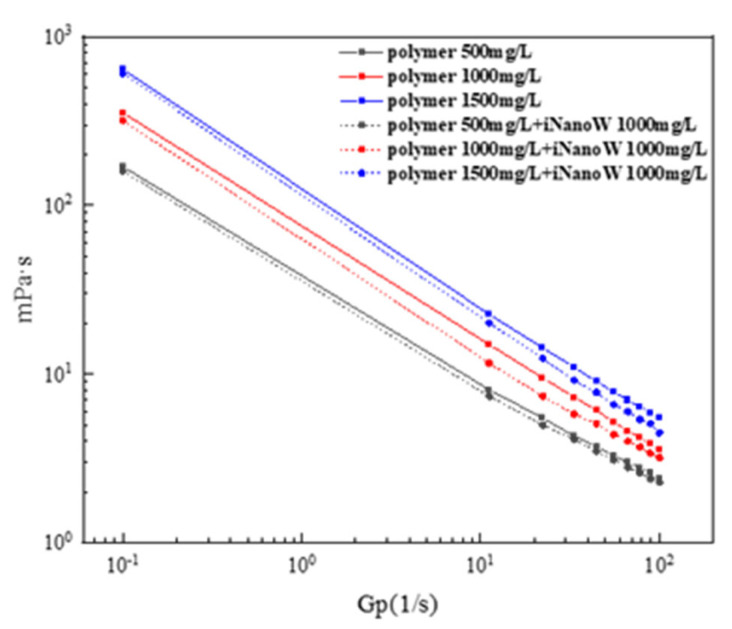
Influence of iNanoW on the Viscosity Behavior of Polymer Solutions.

In this study, we explored the rheological properties of polymer solutions and iNanoW–polymer binary composite solutions through rheological tests, particularly focusing on their apparent viscosity changes at different concentrations and shear rates, as shown in Figure 5. To better quantitatively describe the rheological behavior of polymer solutions at different shear rates, the Power Law model was introduced, and the experimental results are presented in Table 4. As the polymer concentration increased (from 500 mg/L to 1500 mg/L), the flow behavior index (*n*) gradually increased from 0.60 to 0.66, indicating a weakening of shear-thinning behavior. The change in the *n* value reflects the shear flow characteristics of the polymer solution: the smaller the *n* value, the more pronounced the shear-thinning behavior, indicating poorer flow [53,54]. Specifically, as the polymer concentration increased, the hydrogen bonding between polymer molecules became more pronounced, causing more entanglement of polymer chains, which in turn led to an increase in solution viscosity. The specific viscosity changes are reflected in the consistency index (*K*), which increased from 1.59 to 2.10. This change indicates that as the polymer concentration increases, the interaction and entanglement of polymer chains become stronger, resulting in more pronounced shear-thinning behavior and an overall increase in viscosity.

The consistency index (*K*) is an important parameter for describing the viscosity of fluids. An increase in *K* indicates higher viscosity, and this is especially evident in polymer solutions at higher concentrations, where polymer chains become more entangled and the hydrogen bonding enhances the solution’s viscosity [55].

Compared to the pure polymer solution, the flow behavior index (*n*) of the iNanoW–polymer binary composite solution changed significantly, increasing from 0.60–0.69 to 0.61–0.71. This indicates that the shear-thinning behavior became weaker, and the addition of iNanoW reduced the entanglement or interaction between polymer chains. The change in the consistency index (*K*) suggests that iNanoW, by reducing the entanglement of polymer chains, lowers the viscosity of the solution. *K* decreased from 1.59–2.10 to 1.56–2.06, further suggesting that iNanoW can interfere with the hydrogen bonding between polymer chains, reducing their entanglement.

In this study, the change in *K* reflects the increase or decrease in the viscosity of the polymer solution. Lower *K* values indicate a more fluid solution with lower viscosity, suggesting that the addition of iNanoW reduces the viscosity by decreasing the entanglement of polymer chains and improving the rheological properties of the solution.

In summary, the addition of iNanoW significantly affected the hydrogen bonding in polymer solutions. Hydrogen bonding in polymer solutions is a key factor in determining polymer chain entanglement and solution viscosity. As polymer concentration increases, hydrogen bonding becomes stronger, leading to increased interactions between polymer chains and an increase in solution viscosity. However, the addition of iNanoW, by interacting with hydrogen bonds between polymer chains, reduces the entanglement of polymer chains, thereby lowering the viscosity of the solution. Especially at higher concentrations (such as 1500 mg/L), iNanoW significantly reduced the viscosity of the polymer solution, as reflected in the lower *K* values and more pronounced shear-thinning behavior, indicated by the higher *n* values.

### 3.4. Effect of iNanoW on the Surface Tension and Wettability of Polymer Solutions

As shown in Table 5, an increase in polymer concentration leads to a gradual decrease in surface tension and a corresponding reduction in contact angle. This phenomenon is attributed to the polymer’s high molecular weight and hydration effects. As the polymer concentration increases, the interaction between polymer molecules and water molecules strengthens, resulting in more complex polymer chain structures that form a more stable interfacial film. This, in turn, reduces the interfacial tension between the liquid and solid phases, enhancing the wettability of the solution. In our experiments, when the polymer concentration increased from 500 mg/L to 1500 mg/L, the surface tension decreased from 43.0 mN/m to 40.0 mN/m, and the contact angle decreased from 81.3° to 78.1°. This trend aligns with the findings of Ratanpara et al. [56]. and Haghighi [57,58], who reported that increasing polymer concentration typically reduces surface tension, thereby improving wettability. The reduction in contact angle further corroborates this, indicating that the liquid is better able to spread on the solid surface, which contributes to improved oil–water separation efficiency.

Upon the introduction of iNanoW, the surface tension of the polymer solution was reduced, and the wettability was further enhanced. The addition of iNanoW exhibited strong interfacial activity across different polymer concentrations. For instance, at a polymer concentration of 500 mg/L, the introduction of iNanoW decreased the surface tension from 43.0 mN/m to 41.1 mN/m, while the contact angle decreased from 81.3° to 78.0°. As the polymer concentration increased, surface tension continued to decrease, and the contact angle further diminished, indicating that iNanoW effectively improves the wettability and flowability of the solution. This result is consistent with the studies by Sun et al. [32], who found that nanoparticles can adsorb at the oil–water interface, altering the molecular structure and interfacial characteristics, which in turn reduces surface tension and enhances the wettability and flowability of the solution.

However, the presence of iNanoW alone, before the polymer was added, has a higher surface tension (67.3 mN/m) and a larger contact angle (86.8°). This behavior can be attributed to the nanoparticle’s inherent surface energy. While nanoparticles like iNanoW typically have high surface energy due to their large surface area, they tend to exhibit lower wettability when used alone [59]. The reason for the observed decrease in surface tension and contact angle when the polymer is introduced lies in the synergistic interaction between the polymer molecules and nanoparticles. The polymer molecules modify the surface characteristics of the nanoparticles, leading to a more stable interfacial film, which reduces surface tension and enhances wettability.

In summary, the addition of iNanoW improves the interfacial properties of the polymer solution through multiple mechanisms. On the one hand, nanoparticles interact with polymer molecules, altering the conformation of the polymer chains and facilitating the formation of more stable structures at the interface. On the other hand, the interaction between nanoparticles and the liquid interface modifies the adhesion between the liquid and solid surfaces, further enhancing wettability. These changes contribute to the increased permeability and flowability of the polymer solution in core samples, thereby improving the overall efficiency of enhanced oil recovery.

### 3.5. Dynamic Variation of Injection Pressure in Sweep Volume Expansion Core Flooding Experiment

**Figure 6:**
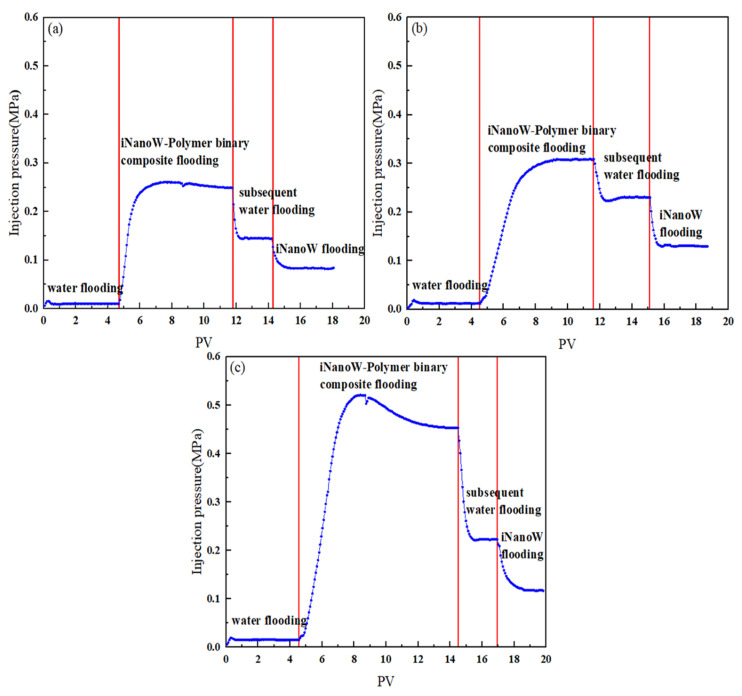
Dynamic Pressure Changes During the Injection Process of iNanoW–polymer binary composite flooding in a 150 mD Core: (**a**) iNanoW (1000 mg/L)-Polymer (500 mg/L) binary composite flooding; (**b**) iNanoW (1000 mg/L)-Polymer (1000 mg/L) binary composite flooding; (**c**) iNanoW (1000 mg/L)-Polymer (1500 mg/L) binary composite flooding.

Figure 6 shows the variation of injection pressure with injection volume (PV) during the iNanoW–polymer binary composite flooding process at different polymer concentrations (500, 1000, 1500 mg/L). All experiments used an iNanoW concentration of 1000 mg/L.

As shown in Figure 6, the injection pressure increases progressively with polymer concentration. This phenomenon indicates that higher polymer concentrations increase flow resistance in the core. The larger the polymer molecular size, the greater the flow resistance in the core, leading to a gradual increase in injection pressure. This is consistent with the properties of polymers, where high-concentration polymer solutions increase fluid viscosity, leading to higher injection pressure.

In the experiment, the final injection pressures of the iNanoW–polymer binary composite flooding segment plugs were 0.249 MPa, 0.308 MPa, and 0.453 MPa, with injection pressure increasing as polymer concentration increased. Specifically, at a polymer concentration of 500 mg/L, the injection pressure was relatively low, while at 1000 mg/L and 1500 mg/L, the injection pressure gradually increased. When switching to subsequent water flooding, the injection pressure significantly decreased. This is because water molecules are smaller, and their flow resistance is lower than that of polymer liquids, leading to a decrease in injection pressure. Water flooding fluids flow more easily than polymer flooding fluids, especially when the injected fluid molecules are smaller, resulting in lower injection pressure. When switching to iNanoW-driven segment plugs, the injection pressure decreased again. This is because iNanoW disrupts hydrogen bonding between water molecules, forming “nanowater” with smaller molecular size than water. “Nanowater” has a smaller molecular size, reducing flow resistance and therefore decreasing injection pressure.

### 3.6. Dynamic Variation of Fluid Distribution in Sweep Volume Expansion Core Flooding Experiment

**Figure 7:**
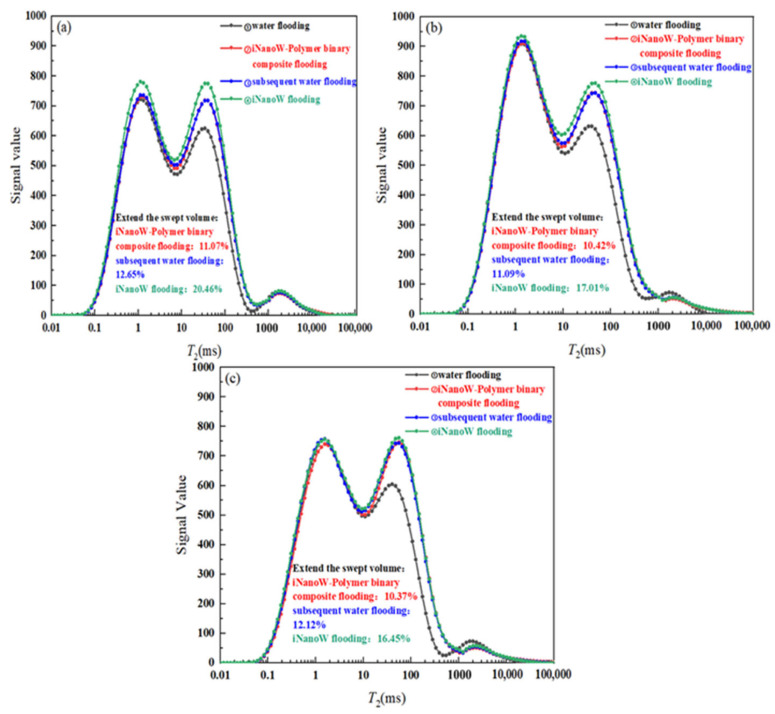
Dynamic Changes in Nuclear Magnetic Resonance *T*_2_ Spectrum During the Injection Process of iNanoW–polymer binary composite flooding in a 150 mD Core: (**a**) iNanoW (1000 mg/L)-Polymer (500 mg/L) binary composite flooding; (**b**) iNanoW (1000 mg/L)-Polymer (1000 mg/L) binary composite flooding; (**c**) iNanoW (1000 mg/L)-Polymer (1500 mg/L) binary composite flooding.

**Figure 8:**
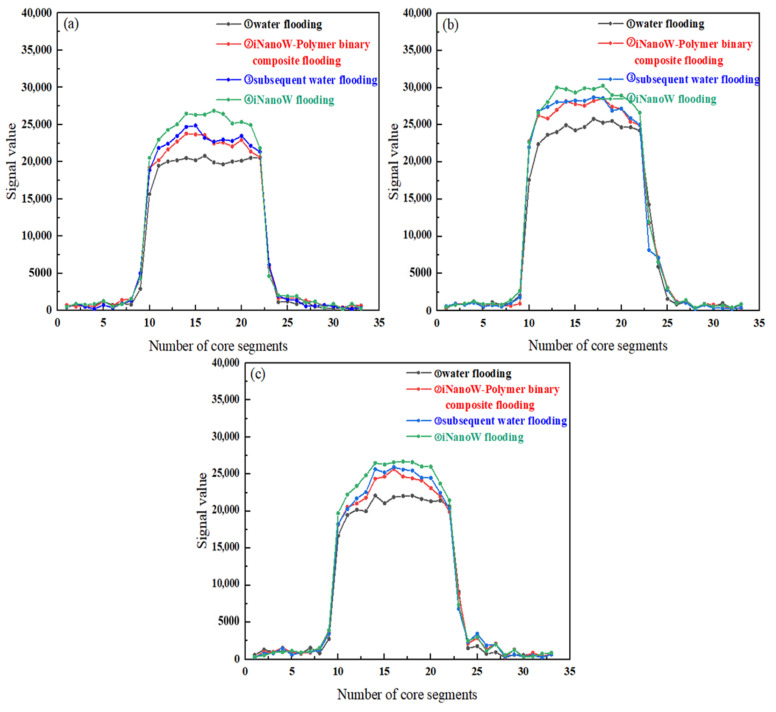
Dynamic Changes in Fluid Distribution within the Core During the Displacement Process of iNanoW–polymer binary composite flooding in a 150 mD Core: (**a**) iNanoW (1000 mg/L)-Polymer (500 mg/L) binary composite flooding; (**b**) iNanoW (1000 mg/L)-Polymer (1000 mg/L) binary composite flooding; (**c**) iNanoW (1000 mg/L)-Polymer (1500 mg/L) binary composite flooding.

Figure 7 and Figure 8 show the *T*_2_ spectra and fluid distribution changes within the core during the iNanoW–polymer binary composite flooding process at polymer concentrations of 500, 1000, and 1500 mg/L (with iNanoW concentration of 1000 mg/L). The sequence of segment plugs in all experiments is: water flooding → iNanoW–polymer binary composite flooding → subsequent water flooding → iNanoW flooding. In Figure 7, the black curve denotes the NMR *T*_2_ distribution following water flooding; the red curve illustrates the spectrum after binary composite flooding using iNanoW and polymer; the green line corresponds to the iNanoW flooding stage; and the blue line represents the subsequent water flooding phase. Within the *T*_2_ distributions, relaxation times ranging from 0.01 to 10 ms are indicative of small pore structures, 10 to 1000 ms correspond to medium-sized pores, and 1000 to 100,000 ms reflect large pore spaces in the core.

Figure 8 depicts the spatial variation of fluid content across different sections of the core during various flooding stages. The core was divided into three areas: segments 9–13 represented the frontal area, segments 14–18 the central area, and segments 19–23 the rear area. Each segment’s plug profile is color-coded to match its corresponding *T*_2_ spectrum for visual correlation.

Figure 7 and Figure 8 show that iNanoW–polymer binary composite flooding significantly expanded the fluid sweep volume in the core compared to water flooding, approximately 10% greater than water flooding. This phenomenon can be attributed to the large size of polymer molecules, as well as the adsorption and retention of polymers in the core’s porous medium. In this way, the polymer creates a pressure gradient within the core, controlling the fluid flow and effectively increasing the fluid content in the front, middle, and rear regions of the core. This effect significantly improves fluid sweep in the medium-pore region, as the polymer can remain in larger pores and push fluids into previously unaffected areas.

When switching to water flooding, although water molecules are smaller and can enter some pores not reached by the iNanoW–polymer binary composite flooding, the sweep volume with water flooding does not significantly exceed the iNanoW–polymer binary composite flooding. Experimental data show that water flooding can only expand the fluid sweep volume in the core by approximately 2%, primarily increasing the fluid content in the front and middle sections of the core. Water molecules, being smaller, can flow through smaller pores, but their sweep effect is relatively limited.

After switching to iNanoW flooding, “nanowater” with smaller molecular size, combined with the flow control of polymers retained during iNanoW–polymer binary composite flooding, allows the fluid to enter more and smaller pores in the core. Therefore, iNanoW flooding further expanded the fluid sweep volume in the core compared to water flooding, with an expansion range of 4.33–7.81%. The effect was particularly noticeable in small and medium pores, significantly improving fluid sweep, and increasing fluid content in the front, middle, and rear regions of the core, with the final sweep effect expanding by 16.45–20.46%.

In summary, iNanoW–polymer binary composite flooding significantly expanded the fluid sweep volume in the core, especially in medium pores. The flow control of polymers allows for more uniform fluid distribution across the core. Although water flooding can penetrate some previously unaffected pores, its effect is relatively limited. iNanoW reduces the size of water molecules, allowing fluids to penetrate more pores, especially in small and medium pores, thereby further expanding the fluid sweep volume. These results indicate that the combination of iNanoW–polymer binary composite flooding, subsequent water flooding, and iNanoW flooding can effectively improve reservoir recovery, particularly in later-stage oilfield development, by optimizing flooding effects and increasing fluid sweep volume, significantly enhancing oil recovery.

### 3.7. Oil Recovery Efficiency Core Flooding Experiment

This section selects the experiment group with the best sweep volume effect from previous experiments to further verify its displacement effect. The sequence of segment plugs is: water flooding → Polymer flooding (500 mg/L)/iNanoW (1000 mg/L)-Polymer (500 mg/L) binary composite flooding → subsequent water flooding → iNanoW flooding. Within the *T*_2_ distributions, relaxation times ranging from 0.01 to 10 ms are indicative of small pore structures, 10 to 1000 ms correspond to medium-sized pores, and 1000 to 100,000 ms reflect large pore spaces in the core.

**Figure 9:**
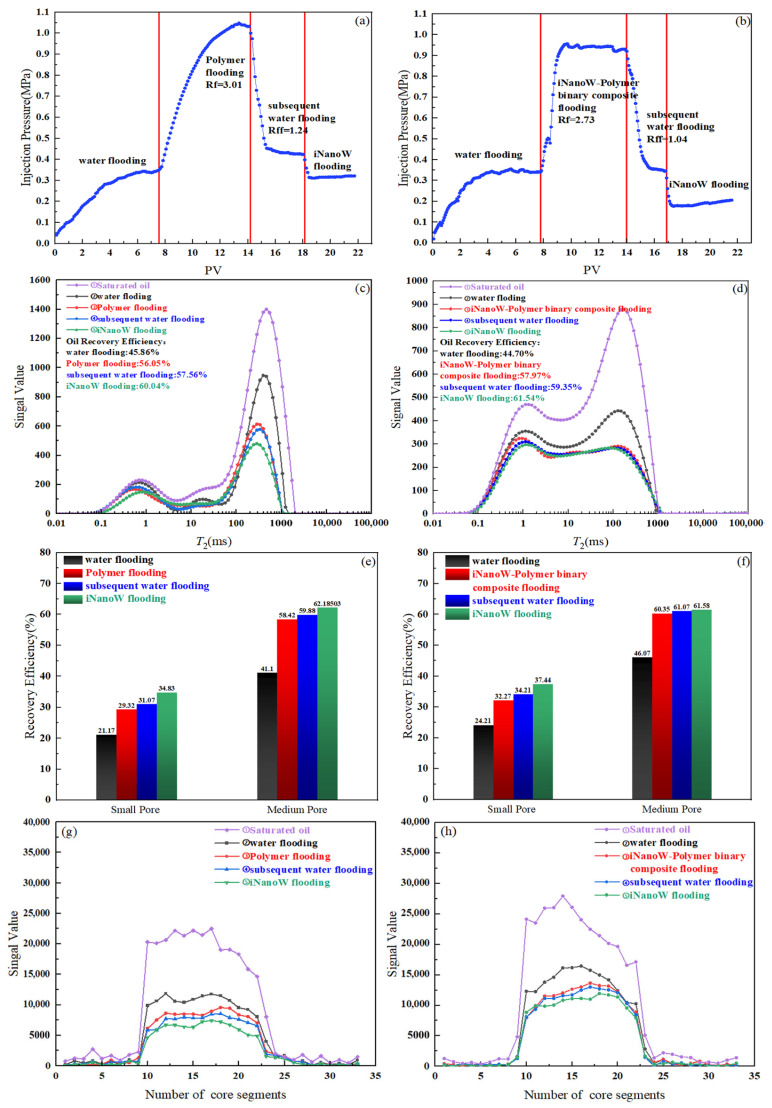
Core Flooding Experimental Results of Polymer flooding and iNanoW–polymer binary composite flooding for Oil Recovery Efficiency in a 150 mD Core: (**a**,**b**) Dynamic Changes in Injection Pressure; (**c**,**d**) Dynamic Changes in *T*_2_ Spectrum; (**e**,**f**) Small/Medium Pores Recovery Efficiency; (**g**,**h**) Dynamic Changes in Fluid Distribution within the Core.

In Figure 9a,b, it can be observed that the injection pressure of the iNanoW–polymer binary composite flooding system is lower than that of the traditional polymer flooding system, with its resistance factor (Rf) being 2.73, significantly lower than the 3.01 observed for polymer flooding. iNanoW reduces the size of polymer aggregates in the solution, which lowers the flow resistance and makes the fluid flow more easily, thereby further enhancing the sweep volume and recovery. The reduction in injection pressure directly improves the fluid displacement efficiency, especially in the iNanoW flooding stage, where the injection pressure is further reduced, optimizing the polymer flooding pressure and the fluid flowability within the pores.

Additionally, surface tension and wettability tests indicate that iNanoW significantly reduces the surface tension of the polymer solution. iNanoW enhances the wettability of the polymer solution, improving its spreading ability on solid surfaces, which helps in better oil displacement, particularly in small pores. The improvement in wettability allows the polymer solution to penetrate more effectively into small pores.

The *T*_2_ spectra provide a clearer view of the residual oil displacement process, especially the distribution in medium and small pores. From Figure 9c–h, the oil recovery efficiency shows that the iNanoW–polymer binary composite flooding system more effectively displaces residual oil in small pores than traditional polymer flooding. Small pores are typically areas that are difficult for traditional flooding methods to fully displace. Specifically, in the polymer flooding process, the oil recovery efficiency for small pores is 29.32%, while in the iNanoW–polymer binary composite flooding, the recovery rate reaches 32.27%. This improvement is attributed to the reduction in aggregate size caused by iNanoW, which allows the polymer to penetrate more small pores and better control flow, thereby improving recovery in these regions. As the water flooding stage progresses, the “nanowater” with smaller water molecule sizes can penetrate more small pores, further displacing residual oil. Ultimately, in the iNanoW–polymer binary composite flooding stage, the oil recovery efficiency in small pores reaches 37.44%.

Overall, the oil recovery efficiency in the iNanoW flooding stage shows significant improvement. Specifically, polymer flooding recovers 56.05% of the oil from medium pores, while the iNanoW–polymer binary composite flooding achieves a recovery rate of 57.97%. This improvement is mainly due to the synergistic effect between the polymer and nanoparticle flooding stages, where “nanowater” penetrates into smaller pores and displaces more oil. iNanoW reduces the size of polymer aggregates, enhancing the permeability and flow control of the polymer, thereby further improving oil recovery efficiency.

**Figure 10:**
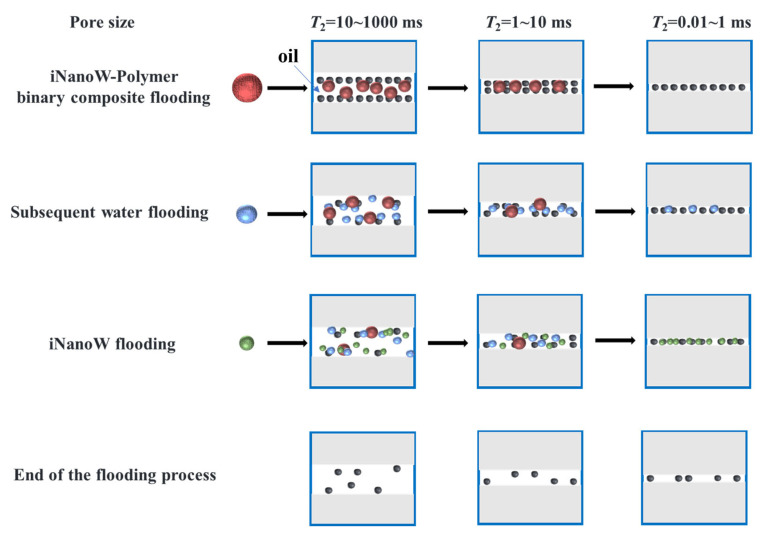
Schematic Diagram of the Flooding Process.

In conclusion, low-field NMR core flooding experiments demonstrate that the iNanoW–polymer binary composite flooding system significantly improves oil recovery efficiency in medium and small pores. The reduced resistance factor, improved fluid dynamics, and the unique properties of “nanowater” molecules allow fluids to penetrate smaller pores more effectively, further enhancing oil recovery efficiency, as demonstrated in Figure 10.

## 4. Conclusions

This study investigates the application of iNanoW nanofluid in a polymer flooding system, particularly its enhanced oil recovery effect in medium-to-high permeability reservoirs. The experimental results indicate that iNanoW weakens the hydrogen bonding between polymer molecules, resulting in a significant reduction in the size of polymer aggregates, ranging from 29% to 36%. This change directly affects the rheological properties of the polymer solution. Rheological analysis based on the power-law model shows that the introduction of iNanoW increases the n value of the polymer solution from 0.60–0.69 to 0.61–0.71, and decreases the K value from 1.59–2.10 to 1.56–2.06, indicating a weakening of shear-thinning behavior and a reduction in viscosity. These changes improve the flowability and injectability of the solution, increase the seepage rate, reduce the Rf value, and enhance the polymer’s permeability and sweep volume in the reservoir. Additionally, the introduction of iNanoW improves the interfacial properties of the polymer solution. Specifically, the surface tension of the polymer solution decreases from 43.0–40.0 mN/m to 41.1–39.4 mN/m, and the contact angle decreases from 81.3–78.1° to 78.0–76.6°, indicating that the introduction of iNanoW significantly improves the wettability of the solution, thereby enhancing oil–water separation efficiency.

In core flooding experiments, the iNanoW–polymer binary composite flooding system significantly expanded the fluid sweep volume. Compared to traditional water flooding, the iNanoW–polymer composite flooding system increased the fluid sweep volume by about 10%, achieving an oil recovery efficiency of 57.97%. More notably, the introduction of iNanoW effectively displaced the residual oil in small pores, with the oil recovery efficiency increasing from 29.32% to 32.27%. This phenomenon indicates that iNanoW, by reducing the size of polymer molecules, improves polymer flowability in the pores, significantly enhancing oil recovery efficiency.

However, in the subsequent water flooding stage, due to the retention of polymer molecules in the pores and the larger size of water molecules, the subsequent water flooding only increased the sweep volume by 1% to 2% based on the iNanoW–polymer composite flooding stage. The sweep effect was relatively limited, mainly concentrated in the areas already displaced by the polymer, and did not effectively displace oil in small pores. In contrast, the iNanoW water flooding stage significantly weakened the hydrogen bonding between water molecules and altered the water network structure, transforming it from “macro-molecule clusters” to “nano-molecule clusters,” forming “nanowater.” This process allowed water to penetrate into small pores that conventional water flooding could not reach, effectively displacing the residual oil in these small pores. As a result, the iNanoW water flooding stage expanded the sweep volume by 20.46%, achieving an oil recovery efficiency of 61.54%, with the recovery rate in small pores reaching up to 37.44%.

In conclusion, the iNanoW-enhanced polymer flooding system demonstrates significant improvement in oil recovery in medium-to-high permeability reservoirs. The introduction of iNanoW enables fluids to access pores that conventional water flooding cannot reach, maximizing the fluid sweep volume and further increasing the recovery. This provides an efficient technological approach to improving oil recovery efficiency in medium-to-high permeability reservoirs, with broad application prospects.

## 5. Prospect

Although this study has demonstrated that the iNanoW–polymer binary composite flooding system significantly improves oil recovery efficiency in medium-to-high permeability reservoirs, there are still multiple aspects that can be further explored and optimized. Below are several potential future research directions:

(1) Optimization of Combinations of Different Nanoparticles and Polymers:

This study primarily focused on the iNanoW–polymer binary composite flooding effect, but future research could explore combinations of other types of nanoparticles (such as metal oxides, carbon nanotubes, etc.) with different types of polymers. By systematically comparing the properties of various nanoparticles and their synergistic effects with polymers, the composite flooding system can be optimized to improve oil recovery efficiency.

(2) Distribution and Migration Behavior of Nanoparticles in Pores:

The distribution, migration, and stability of iNanoW in reservoirs still need further study. Particularly in complex core structures, how nanoparticles interact with oil, water, and polymers and maintain uniform distribution in the pores is crucial for the final displacement effect. Advanced imaging techniques (such as CT scanning, nuclear magnetic resonance, etc.) can be used to track the movement and distribution of nanoparticles in the pores, providing deeper insights into nanoparticle flow behavior.

(3) Further Study on the Synergistic Mechanism between Polymer and Nanoparticles:

The interaction mechanism between polymers and nanoparticles remains a key issue. Future research can explore the specific interactions between polymer chains and nanoparticles (such as hydrogen bonding, electrostatic interactions, etc.) through molecular dynamics simulations and surface science analyses. This will help reveal how these interactions affect the rheological properties, wettability, and oil–water separation efficiency of polymer solutions.

Future research can delve into various aspects of the iNanoW–polymer binary composite flooding technology’s potential, including optimizing the selection of nanoparticles, and gaining a deeper understanding of the interactions between polymers and nanoparticles. Through these studies, oil recovery efficiency can be further enhanced, pushing oilfield development technology towards more efficient and sustainable directions.

## Figures and Tables

**Table 1:** Basic Parameters of Core for Experiment.

Core	Length	Diameter	Porosity	Gas Phase Permeability	Water Phase Permeability
NO.	/cm	/cm	/%	/mD	/mD
150-1	5.05	2.52	14.21	150.52	16.23
150-2	5.03	2.53	13.99	149.87	16.95
150-3	5.01	2.51	14.16	151.02	16.98
150-4	5.02	2.52	14.07	152.24	16.08
150-5	5.03	2.5	14.32	152.24	16.24
150-6	5.04	2.51	14.54	151.23	16.32
150-7	5.02	2.53	14.25	152.25	15.98
150-8	5.01	2.49	14.27	150.88	16.32
150-9	5.00	2.5	14.3	151.21	16.24
150-10	5.02	2.52	14.22	152.42	15.99

**Table 2:** The Formulation of the iNanoW–polymer Binary Composite Solution.

iNanoW–Polymer Binary Composite Solution	Polymer Mass Concentration	iNanoW Mass Concentration	QY-7	iNanoW Nanofluid	Brine
	mg/L	mg/L	/g	/g	/g
Formula (1)	500	1000	0.147	0.500	99.353
Formula (2)	1000		0.293		99.207
Formula (3)	1500		0.440		99.06

**Table 3:** Experimental Results of Injectivity Evaluation.

Core	Polymer Mass Concentration	Flooding System	Seepage Time	Flow Rate	Seepage Velocity	Injection
			t	Q	V	
NO.	(mg/L)		(min)	(mL/min)	(mL/min)	
150-1	500	polymer flooding	31.6	0.0158	0.023	Good
150-2	1000		52.1	0.0096	0.014	Mid
150-3	1500		80.6	0.0062	0.009	Poor
150-4	500	iNanoW–polymer binary flooding	28.1	0.0178	0.026	Good
150-5	1000		40.65	0.0123	0.018	Mid
150-6	1500		65.8	0.0076	0.011	Poor

**Table 4:** Experimental Results of Consistency Index (*K*) and Behavior Index (*n*).

Flooding System	Polymer Mass Concentration	iNanoW Mass Concentration	*n*	*K*
	mg/L	mg/L		
Polymer flooding	500	0	0.60	1.59
	1000		0.65	1.88
	1500		0.69	2.1
iNanoW–polymer Binary Composite flooding	500	1000	0.61	1.56
	1000		0.67	1.81
	1500		0.71	2.06

**Table 5:** Experimental Results of Surface Tension and Contact Angle Evaluation.

Solution	Polymer Mass Concentration	iNanoW Mass Concentration	Surface Tension	Contact Angle
	mg/L	mg/L	mN/m	°
Polymer solution	500	0	43.0	81.3
	1000		41.7	79.5
	1500		40.0	78.1
iNanoW–polymer Binary Composite solution	500	1000	41.1	78.0
	1000		40.1	77.5
	1500		39.4	76.6
iNanoW solution	0	1000	67.3	86.8

## Data Availability

Data are contained within the article.
